# Non-just-right experiences are more closely related to OCD than tics in Tourette patients

**DOI:** 10.1038/s41598-023-37658-0

**Published:** 2023-11-10

**Authors:** Valerie Brandt, Jan-Hendrik Otte, Carolin Fremer, Ewgeni Jakubovski, Kirsten Müller-Vahl

**Affiliations:** 1 Clinic of Psychiatry, Social Psychiatry and Psychotherapy, Hannover Medical School, Hanover, Germany; 2 School of Psychology, Centre for Innovation in Mental Health, University of Southampton, Southampton, UK

**Keywords:** Psychology, Medical research

## Abstract

Complex tics and obsessive or compulsive behaviour can be difficult to differentiate diagnostically. The majority of adult patients with Tourette syndrome report experiencing premonitory urges before tics. Some of these experiences have been linked to non-just-right experiences (NJRE), which are frequently reported by patients with obsessive–compulsive disorder or behaviours (OCD/OCB). We aimed to assess whether NJRE are more closely related to tics and tic-associated premonitory urges or whether they are more closely associated with OCD. A total of N = 111 patients (mean age = 34.77 + /−12.93; N = 37 female) with a confirmed diagnosis of Tourette syndrome completed the premonitory urges for tic disorders scale (PUTS), the revised non-just-right experiences scale (NJRE-QR), and questionnaires regarding their tic severity, and comorbid OCD/OCB. A multi-trait-multi-methods matrix was calculated to examine associations amongst scales measuring tic-related and OCB-related phenomena. The PUTS correlated overall higher with tic questionnaires than with OCD/OCB questionnaires. The NJRE correlated higher with OCD symptoms than with tic severity. The results indicate that non-just-right experiences are more closely associated with comorbid OCB than with tics in patients with Tourette syndrome.

## Introduction

Tourette syndrome (TS) and chronic tic disorders are characterized by multiple motor and/or vocal tics^1^. The majority of adults report that they experience a premonitory urge (PU), a sensation that precedes tics^2–6^. PU tend to increase before a tic and then decrease in the majority of patients after a single tic or a bout of tics has been executed^7,8^. However, patients can experience a variety of different patterns^8^. Adults with TS report PU more often than children. Approximately 77% of patients over 13 years report that they experience PU, while approximately 90% of patients over 18 years report to experience PU. It is therefore unclear whether urges develop as a consequence of tics or precede tics in development. It is possible that young children may lack awareness or the ability to describe PU^9^.

In patients with tics, PU tend to occur at the same place where tics occur^10^, most often in the face and head^10^. Furthermore, PU have been found to be associated with lower quality of life^6,11^. Urges of different qualities have been described, such as a feeling of pressure or not-just-right experiences (NJRE)^9,12^. NJREs refer to the feeling that something within oneself or the surroundings is not quite as it should be. While urges in general are very common in patients with TS, the most common feeling is a feeling of tension, while specific sensations, such as NJREs, are less common and seems to occur in approximately a third of patients with chronic tic disorders^13^. NJREs appear to be associated more frequently with other symptoms than tics^14^.

Patients with TS who seek diagnosis or treatment typically have at least one comorbidity, the most common are attention deficit/hyperactivity disorder (ADHD), obsessive–compulsive behavior/disorder (OCB/OCD), depression, and anxiety^15^. OCD is defined by obsessions, i.e., “recurrent and persistent thoughts, urges, or images", which are unwanted and cause distress, and compulsions, i.e., repetitive external or internal behaviors that are performed in response to an obsession^1^. While urges are considered typical of tics, they can also precede compulsions instead of obsessions in OCD^14,16^, specifically NJRE might occur in at least 70–80% of patients with OCD^16,17^. NJREs and feelings of incompleteness are considered two of the drivers of OCB^18^. NJREs have also been associated with comorbid OCD in TS^19^. This study was conducted to investigate whether NJREs are more closely related to tics and tic-associated premonitory urges or whether they are more closely associated with OCB in patients with chronic tics.

## Methods

A total of N = 111 patients were recruited from the Tourette syndrome specialty clinic at the Hannover Medical School (mean age = 34.77 + /−SD = 12.93; N = 37 female (33%)). Patients who were at the clinic for either a clinical routine assessment or for participation in another study^20,21^ were recruited to fill out the questionnaires. Patients were only included if they had a confirmed diagnosis of TS. Patients at this clinic are diagnosed by an experienced psychiatrist/neurologist (KMV) according to DSM-5 criteria^22^. Demographic information and mean questionnaire scores of the sample are displayed in Table 1.

**Table 1:** Demographic data.

	N	Range	Possible range
Sex	37 F/74 M	N/A	N/A
	M (SD)	Range	Possible range
Age	34.8 (12.9)	18–73	N/A
YGTSS-TTS	24.5 (8.6)	8–46	0–50
YGTSS Impairment	23.4 (12.5)	0–50	0–50
YGTSS-GSS	47.9 (18.1)	12–87	0–100
ATQ	46.9 (23.4)	8–102	0–108
PUTS_9_	20.7 (6.0)	9–35	9–36
TS-QoL	25.9 (16.9)	1–74	0–108
Y-BOCS	5.1 (7.5)	0–28	0–40
NJRE-QR	20.9 (11.2)	7–48	9–56
USP-SPS	5.7 (4.2)	0–15	0–15
FSU-12	9.9 (10.7)	0–43	0–48
OC-TDCQ	14.0 (9.5)	0–37	0–80
BDI-II	12.4 (11.0)	0–50	0–63
BAI	12.0 (10.3)	0–41	0–63

Table displays age and sex data, as well as means (M) and standard deviations (SD), the range, and the possible range for the questionnaires used in the study. These include the Yale Global Tic Severity Scale total tic severity score (YGTSS-TSS), the YGTSS global severity score (YGTSS-GSS = YGTSS-TSS + impairment), the Adult Tics Questionnaire (ATQ), the Premonitory Urges for Tic Disorders Scale (PUTS), the Tourette Syndrome Quality of Life (TS-QoL) scale, the Yale-Brown Obsessive Compulsive Scale (Y-BOCS), the revised Non-Just-Right-Experiences Questionnaire (NJRE-QR), the University of Sao Paulo Sensory Phenomena Scale (USP-SPS), the Feelings of Incompleteness Questionnaire (FSU-12), the Obsessive–Compulsive Trait Core Dimensions Questionnaire (OC-TCDQ-R), Becks Depression Inventory (BDI-II), and Becks Anxiety Inventory (BAI).

The study was reviewed and approved by the Hannover Medical School’s ethics committee (no. 7631) and adhered to the declaration of Helsinki. Patients gave written informed consent to participate in the study.

### Measures

Patients filled out a number of questionnaires including the University of Sao Paulo Sensory Phenomena scale (USP-SPS) assessing a range of sensory phenomena^23^, the German version^24^ of the Revised-Non-Just-Right-Experiences Questionnaire (NJRE-QR) assessing NJREs^25^, and the Premonitory Urge for Tic Disorders scale (PUTS)^12^ assessing different PU phenomena related to tics. Previous validations of the PUTS have shown that the last item suffers from suboptimal psychometric properties^19,26,27^ and is therefore commonly excluded. We are therefore also excluding this item from our analyses and will refer to this version as the PUTS_9_. Tic severity was assessed by the clinician-rated Yale Global Tic Severity Scale (YGTSS), for the analyses the total tic score (TTS) was used^28^. Additionally, the self-assessment Adult Tics Questionnaire (ATQ) was used to assess tic severity^29^ as well as the Tourette syndrome Quality of Life Scale (GTS-QoL) to measure the disease-related quality of life^30^. Severity of OCB/OCD was assessed using the self-assessment Obsessive–Compulsive Trait Core Dimensions Questionnaire (OC-TCDQ-R) which focuses on harm-avoidance and incompletness^31,32^, the self-assessment Feelings of Incompletes Questionnaire (FSU-12)^33^, and the clinician-rated Yale Brown Obsessive Compulsive Scale (Y-BOCS)^34,35^. The YGTSS and the Y-BOCS are considered the gold standard for assessing tics and comorbid OCD symptoms in patients with Tourette syndrome, according to the European Society for the Study of Tourette Syndrome (ESSTS) guidelines^36^. Comorbid depression and anxiety symptoms were assessed using the revised version of the Becks Depression Inventory (BDI-II)^37^, and the Becks Anxiety Inventory (BAI)^38^. Both are commonly used and well validated self-report measures. Possible ranges of the scales are reported in Table 1.

### Statistical analysis

An a-priori power calculation in G*Power showed that a sample size of N = 96 was necessary to find a medium sized effect if there was one. Achieved power with the sample size of N = 111 was 1−ß = 0.97.

NJRE-QR was calculated for different comorbidities. Clinically relevant OCD was defined as YBOCS ≥ 16, clinically relevant depressive and anxiety symptoms were defined as at least moderate symptoms (BDI-II ≥ 20; BAI ≥ 16). Independent t-tests were conducted for each group and compared to a group with none of the three comorbidities. However, each patient group with a comorbidity was not independent from the other comorbidity groups, i.e., a patient with clinically relevant OCD could also score above the cut-off for depression and would be part of the OCD and depression comorbidity group. A Pearson’s correlation between the number of comorbidities and NJRE intensity was therefore also calculated.

In order to assess how closely similar and different constructs were related, we conducted a multi-trait-multi-methods matrix (MMTM). MMTMs are typically used in questionnaire validation to show whether a construct measured using a questionnaire is similar to another questionnaire measuring the same construct or trait (multiple methods to measure the same trait) and whether questionnaires assessing different constructs show lower correlations (suggesting multiple different traits were assessed). Correlations were conducted using Spearman’s rank correlations (rho).

## Results

**Figure 1:**
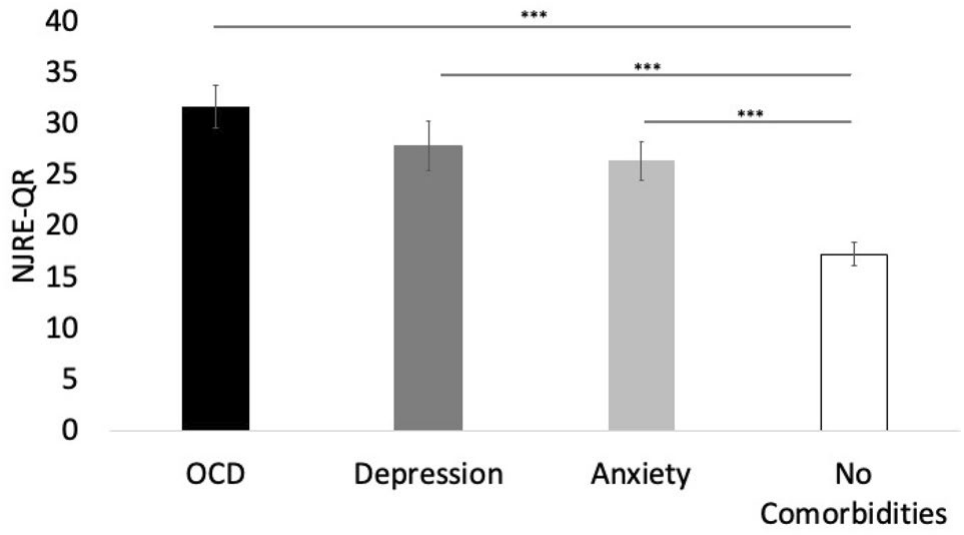
Patients with chronic tics and comorbid obsessive–compulsive disorder (OCD) showed the highest mean score on the Non-Just-Right-Experiences Questionnaire (NJRE-QR), significantly higher than patients with chronic tics without clinically significant OCD symptoms. Patients with clinically significant depression and anxiety symptoms also showed significantly higher NJRE-QR scores than patients without clinically relevant depression or anxiety symptoms. Error bars are standard errors.

Patients who participated had never received psychotherapy (in particular “habit reversal training”, a form of cognitive behavioural therapy (CBT) specifically for treating tics) for the treatment of their tics. Those patients that were taking medication had been on stable medication for at least 6 weeks. A total of N = 13 patients had clinically relevant OCD according to the Y-BOCS (cut-off = 16), N = 22 had moderate or severe depression (BDI-II ≥ 20), and N = 33 had moderate or severe anxiety (BAI ≥ 16), while N = 69 had none of the assessed comorbidities. The mean NJRE-QR for each comorbidity is reported in Fig. 1. The number of comorbidities was significantly correlated with NJRE severity (*r* = 0.43, *p* < 0.001).

Spearman’s rank correlations show that all questionnaires assessing PU (PUTS_9,_ NJRE-QR, and the USP-SPS) correlate in the medium range (Table 1). We calculated the total PUTS_9_ score and correlated it with the NJRE-QR. In order to avoid autocorrelation, we removed item 4 of the PUTS from the total score as it measures NJREs (the same construct as assessed by the NJRE-QR). Correlation between the PUTS_9-item4_ (without the NJRE item 4), and NJREs measured with the NJRE-QR also correlate in the medium range (*rho* = 0.34, *p* < 0.001). Even item 4 of the PUTS asking specifically about NJREs in patients with tics correlated with NJREs as measured by the NJRE-QR only in the medium range (*rho* = 0.33, *p* < 0.001), indicating that the underlying concepts differ somewhat. Correlations between tic severity and premonitory urges were small when measured with the YGTSS-TTS, and medium-sized when measured with the ATQ. Substantial correlations were found between NJREs (NJRE-QR), harm-avoidance and incompleteness (OC-TCDQ), as well as feelings of incompleteness (FSU-12) (Table 2).

**Table 2:**
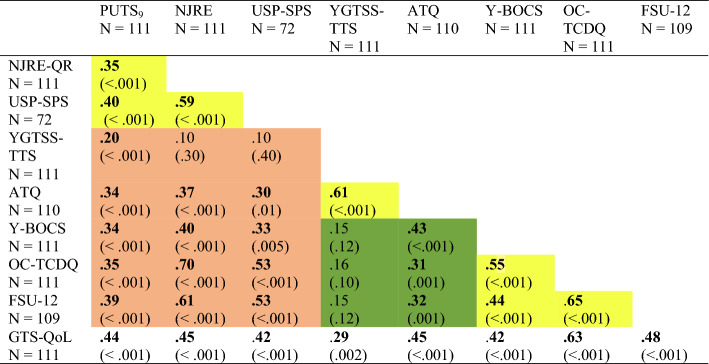
Multi-trait-multi-methods matrix.

The table displays Spearman’s rho correlations in a multi-trait-multi-methods matrix. Yellow (light) shading represents same traits or concepts, measured with different questionnaires. Brown (darker) shading reflects different but related concepts (e.g., urges and tics), and green (dark) shading reflects different concepts or traits (e.g., TS and OCD). Information for quality of life (GTS-QoL9) was also added.

NJRE-QR = Revised-Non-Just-Right-Experiences Questionnaire; USP-SPS = University of Sao Paulo Sensory Phenomena scale; YGTSS-TTS = Yale Global Tic Severity Scale Total Tic Score; ATQ = Adult Tics Questionnaire; Y-BOCS = Yale Brown Obsessive Compulsive Scale; OC-TCDQ-R = Obsessive–Compulsive Trait Core Dimensions Questionnaire; FSU-12 = Feelings of Incompletes Questionnaire; GTS-QoL = Tourette Syndrome Quality of Life Scale.

## Discussion

The results show that questionnaires that measure the same construct correlated quite highly with one another, as would be expected. This shows convergent validity between questionnaires that are expected to assess the same underlying construct. This was true for the YGTSS-TTS and the ATQ, both questionnaires assessing tic severity, and this is in line with previous research showing high correlations between the two questionaires^29,39^.

A high correlation was also found between the OC-TCDQ and the FSU-12 questionnaires, both assessing, at least in part, feelings of incompleteness^31,33,40^. Further relatively high correlations were found between the OC-TCDQ and the Y-BOCS, assessing OCB, suggesting an overlap of the measured underlying concept.

There were medium correlations between the PUTS_9_ and the YGTSS-TTS as well as the ATQ, as would be expected for questionnaires that assess distinct but related constructs^12,26,41,42^. However, correlations between the PUTS_9_ and measures of OCB/OCD (Y-BOCS, OC-TDCQ, and FSU-12) were in the same range. It should be noted that the sample only contained patients with TS and contained no patients with OCD only (without tics); out of the sample, only 13 patients scored in the clinical range regarding their OCB. It is therefore possible that in patients with TS premonitory sensations are quite closely related to OCB.

The NJRE-QR on the other hand, distinguished better between OCB and tics. Correlations between the NJRE-QR and questionnaires assessing OCB were medium–high, while the correlation between the NJRE-QR and the YGTSS-TTS was small (though in the medium range with the ATQ). The correlation between the NJRE-QR and the PUTS_9-item4_ (PUTS_9-_without the item 4 asking specifically about NJREs in patients with tics) was in the medium range. It appears that NJREs are related to other premonitory sensations, but NJREs are more closely linked with OCB than they are with tics. Premonitory sensations and NJREs cannot be considered the same construct but they are related.

Furthermore, NJREs correlated with the number of comorbidities and was higher in patients with comorbid OCD than in patients with comorbid anxiety or depression, although these were also significantly higher than in patients without those comorbidities. This is in line with previous research showing NJREs in OCD, eating disorders, hair pulling disorder, and gambling disorder^17^. NJREs appear to be quite common across disorders but are particularly frequently associated with OCD symptoms.

There was a medium correlation between the Y-BOCS and the ATQ, indicating that the ATQ did not differentiate well between tic severity and severity of comorbid OCD symptoms. This was also reflected in a medium correlation between the ATQ and the NJRE-QR, which was not much lower than the correlation between the NJRE-QR and the Y-BOCS. This is somewhat in contrast to a previous study, showing lower correlations between the ATQ and the Y- BOCS and suggests that the psychometric properties of the ATQ should be more thoroughly tested^29^.

### Strengths and limitations

Strengths of this study include a large patient population with a confirmed tic disorder diagnosis, no prior experience with psychotherapy for tics, and the use of self-rated as well as clinician rated instruments. Weaknesses include that relatively few patients had comorbid OCD and that most included patients had mild OCB. Most patients who participated were seeking treatment. Moreover, the USP-SPS was only available for N = 79 patients, however, the questionnaire was not part of the main hypothesis of this study. Otherwise, there were very few patients missing, and those patients that filled in questionnaires had complete datasets.

By now, new, adapted versions of the PUTS (the iPUTS^43^ and the PUTS-R^26^) have been developed but only after ethics for this project was submitted, hence, they were not used in this study.

## Conclusions

NJREs are experienced by some patients with TS but are more closely linked with symptoms of OCB/OCD than with tics in patients with Tourette syndrome and other chronic tic disorders. Tics and compulsions can be difficult to differentiate, especially when compulsions are associated with urges instead of obsessions. Experiencing NJREs could be considered an indicator that a behaviour is more likely compulsive than a tic.

## Data Availability

The data presented in this study are available on request from the senior author (K.M.-V.).
